# Effects of a Psychoeducational Program on Hemoglobin A1c Level and Health-Related Quality of Life in Patients with Type 2 Diabetes Mellitus, Jazan, Saudi Arabia

**DOI:** 10.1155/2018/6915467

**Published:** 2018-05-14

**Authors:** Samy Shaban Mahmoud, Mona Husein EL Mahdy, Mohamed Salih Mahfouz, Ibrahim Saad Nada, Abdulwahab Abdoh Aqeeli, Mohammed Ahmed AL Darbi, Anas Elias Ahmed

**Affiliations:** ^1^Department of Family and Community Medicine, Faculty of Medicine, Jazan University, Jazan, Saudi Arabia; ^2^Department of Community and Occupational Medicine, Faculty of Medicine, Al-Azhar University, Cairo, Egypt; ^3^Department of Community Medicine, Faculty of Applied Medical Sciences, Umm Al-Qura University, Mecca, Saudi Arabia; ^4^Department of Family Medicine, Ministry of Health, Jazan, Saudi Arabia

## Abstract

**Background:**

Diabetes mellitus (DM) is a growing health problem. Care programs should involve the patients to upgrade their diabetes condition and health-related quality of life (HRQoL).

**Objective:**

The present study aimed to assess the effects of a psychoeducational intervention program on an indicator of glycemic control and HRQoL among type 2 diabetic patients.

**Methods:**

In this quasi-experimental (pre- and postinterventional) study, 99 outpatients with type 2 diabetes were selected randomly from those attending primary health care centers in Jazan, Saudi Arabia, in 2016. Hemoglobin A1c levels (HbA1c) were measured by the colorimetric method, and HRQoL was assessed by the Arabic version of the RAND 36-Item Health Survey 1.0 (RAND-36). The psychoeducational program was conducted on the participants for 4 weeks, and preprogram findings were compared with the postprogram findings after a 5-month follow-up.

**Results:**

After the intervention, there was a statistically significant reduction in the mean value of HbA1c from 9.8 to 7.7 (*P* < 0.001), and there was significant improvement in the mean scores of the following HRQoL scales: role limitations due to emotional problems, energy/fatigue, emotional well-being, and general health (*P* < 0.01). In addition, the impact of the program on HRQoL was better among males and among patients who were older than forty years than among women and patients who were forty years old or younger.

**Conclusion:**

The application of such psychoeducational intervention programs can be helpful in the improvement of HbA1c levels and HRQoL for patients with DM.

## 1. Introduction

Diabetes mellitus (DM) is defined as a group of metabolic diseases characterized by hyperglycemia that results from defects in insulin secretion, insulin action, or both [1]. According to the International Diabetes Federation, 285 million people are affected by diabetes globally, and the number is expected to increase to 438 million by the year 2030, with two-thirds of all cases of DM occurring in developing countries. The number of adults with impaired glucose tolerance is expected to rise from 344 million in 2010 to 472 million by 2030 [2], which reflects the increase in predisposing risk factors, such as obesity or overweight [3].

Regarding the situation of DM in Saudi Arabia, a study published in 2011 revealed that the prevalence of type 2 diabetes was 30% in males and 27.6% among females [4]. Moreover, a community-based study conducted between 1995 and 2000 in Saudi Arabia reported that the overall prevalence of type 2 DM was 23.7%, with a prevalence in males and females that was 26.2% and 21.5%, respectively [2].

Diabetes might have an adverse effect on the patients' health in general and on their quality of life (QoL) in particular. Several complications might occur with long-term diabetes, such as microvascular complications (e.g., retinopathy and neuropathy) and macrovascular complications (e.g., myocardial infarction, angina pectoris, and stroke) [5, 6]. These complications lead to a significant reduction of patients' HRQoL [7].

For optimum control of diabetes, patients must know the importance of their diet and medication and be familiar with ways to modify them according to their exercise routine [8]. Hence, diabetes self-care education is a core element of diabetes management [9]. The findings of several studies indicated the positive impact of diabetes self-care educational interventions on patients' HRQoL [10].

Unfortunately, less than 50% of diabetic patients have adequate knowledge and gain the needed skills for control of their diabetes, and the optimum glycemic control (HbA1c < 7.0%) is achieved only by less than 50% of type 2 diabetics [11].

Briefly, DM educational programs have been suggested for improving the patients HRQoL [12]. Moreover, increasing evidences suggest that DM education programs that address the self-care and the psychological care of diabetic patients improve their QoL and help them in controlling their diabetes [13]. Although several studies have been conducted in Saudi Arabia addressing the prevalence, risk factors, and care for DM [14, 15], few studies addressed HRQoL among diabetics and to the best of our knowledge no study has assessed the impact of educational programs on HRQoL among diabetics in Jazan. Thus, the aim of this study was to assess the effects of a psychoeducational intervention program on HbA1c levels and HRQoL in patients with type 2 diabetes in Jazan.

## 2. Materials and Methods

### 2.1. Study Design, Settings, and Participants

This research work was a quasi-experimental (pre- and postinterventional) study which was conducted on a sample of diabetic patients in the city of Gizan, the capital of Jazan Province, Saudi Arabia. The region is located in the Southwest of Saudi Arabia and is north of Yemen. Its population consists of 1.5 million people. The study was conducted from August 2016 through March 2017 in four primary health care centers (PHCCs) in Gizan. Diabetic patients who fulfilled the inclusion criteria were identified and included in the study. The inclusion criteria were specifically as follows: (1) patients who were diagnosed with type 2 diabetes and were receiving oral hypoglycemic and/or insulin therapy; (2) patients who provided written informed consent and expressed a willingness to abide by the rules of the study; (3) those who had not attended another formal diabetes education program; (4) patients who continued during the study program on the same DM drug regimen prescribed at the study entry. Exclusion criterion included patients who were younger than 18 years.

### 2.2. Sampling Strategy

The sample size was calculated using the *t*-test statistical formula [16] for the difference between two dependent means (matched pairs), which is written as follows: *n* = (*t*_(*n* − 1/(*α*/2))_ + *t*_(*n* − 1, *β*)_)^2^/*d*^2^, where *n* is sample size, *d* is the effect size/sd, *α* is alpha, and power = (1 − *β* error prob). Using the effect size = 0.3, *α* = 0.05, power (1 − *β* error prob) = 0.8, and sd = 1, the initial sample size was estimated at 90 diabetic patients. However, after accounting for a 10% nonresponse rate, the final sample size was increased to 100 diabetic patients. For the implementation of the study, four PHCCs were selected using stratified random sampling technique. Stratification was based on the accreditation status of the 12 PHCCs in Jazan city. Two centers were randomly selected from the five accredited PHCCs, while two others were selected from the seven unaccredited centers. Twenty-five patients were randomly selected from each selected PHCC as the attendance rates were nearly the same.

### 2.3. Intervention

The psychoeducational intervention program was designed according to protocol of the Saudi Diabetes & Endocrine Association. The main objectives of this program were as follows: (a) to upgrade and enhance patients' understanding about the nature and causes of diabetes, (b) to provide information on the complications of diabetes mellitus and to teach patients the standards of medical and nutritional care, (c) to encourage self-care, lifestyle modifications, and good compliance with medication, and (d) to help patients accept living and working with DM.

This psychoeducational program was based on a variety of interactive educational methods and techniques involving counseling [17], demonstration [18], group discussion [19], and vignettes [20]. Education sessions were held at the chronic disease clinic for three hours weekly for four weeks, and then participants were followed for five months. The program covered the following areas: a diabetes overview and its complications, self-care, medications and their side effects, lifestyle modification, clarification of myths and misconceptions, and coping skills for living with diabetes. Participants were classified into 10 groups, and the educational class was held by one psychoeducator and one nurse for each group. During classes, patients were encouraged to speak freely, ask their questions, express their feelings, exchange their own experiences, and receive feedback from the group members and instructors. After the psychoeducational sessions, participants were followed for 5 months, and then HRQoL and HbA1c were reassessed to determine the effects of the program intervention on them.

### 2.4. Instruments and Data Collection Techniques

Information on demographic characteristics, diabetes-related history, type of DM drug regimen, preexisting medical conditions, and HRQoL was collected through a formal interview. The HRQoL was measured by using the validated and reliable Arabic version of the RAND-36 [21]. In addition, blood samples were taken for measuring HbA1c in laboratories of the PHCCs. HbA1c was tested by colorimetric method using hemolysate (BioSystems, Barcelona, Spain) and was measured twice: before the psychoeducational intervention and 5 months after intervention. Type of treatment was assessed before and after the intervention to ensure that patients involved in the program did not change their drug regimens prescribed at the study entry.

The RAND-36 is a generic HRQoL measure emanated from work begun at RAND Corporation in 1984 as part of Medical Outcomes Study (MOS) [22, 23]. Its validity was assessed and ensured among different populations [24, 25]. The Arabic version of the RAND-36 was examined during the translation process by experts and committee members and by pretesting and cognitive debriefing to ensure content validity [21]. For ensuring the reliability of our study tool, a pilot study involving 30 patients who were not included in the survey was conducted to assess the internal consistency reliability; Cronbach's coefficient alpha was used to estimate internal consistency reliability coefficients for the initial and the retest tool administrations. It revealed that the median Cronbach's alphas for the tool (initial and retest) exceeded 0.82 for all scales except for the general health scale (the median Cronbach's alpha = 0.69). Moreover, test-retest reliability was assessed and Pearson product-moment correlations were computed between the initial and the retest administrations to assess the test-retest reliability over a two-week interval. It showed that correlation coefficients (*r*) > 0.70 (with *P* < 0.01 for all).

### 2.5. Statistical Analysis

Data were collected and coded and then analyzed and tabulated using the Statistical Package for Social Science (SPSS version 20, IBM, Chicago, USA). Descriptive statistics were used: frequencies and percentages for the categorical data and means and standard deviations for the quantitative data. Two-sample comparisons were made by using Student's *t*-tests. Normality assumptions were assessed before parametric tests were used. Reliability was assessed using Cronbach's alpha coefficient. The main outcome variables were the mean scores of HRQoL scales and the mean values of HbA1c as they were compared before and after the implementation of the psychoeducational program. A *P* value less than 0.05 was used as a cutoff point to indicate statistical significance.

### 2.6. Ethical Considerations

The study instruments and protocols were approved from the IRB committee of the Faculty of Medicine, Jazan University (IRB number: 1436-SCBRE-27). Informed consent was obtained from all individual participants included in the study. During the study program, if there was any change occurring in patients' initial characteristics which could affect the outcome variables, patients would have been allowed to participate but their data would be excluded from the analysis. The data were handled and stored in accordance with the tenets of the Declaration of Helsinki (1964, amended in 2008).

## 3. Results

The response rate of the study was 99% (99 from the target of 100 participants). The baseline data for all participants indicated that 58.6% were older than forty years, 57.6% were males, 58.6% were married, 42.4% were illiterate, and 66.7% came from rural areas. However, 46.5% were on combination therapy (insulin + oral hypoglycemic tablets), 38.4% were on oral hypoglycemic tablets, and 15.2% were on insulin. The mean duration of diabetes was 8.25 (SD 3.47) years, and 60.6% were complicated diabetics. In addition, the mean level of HbA1c was 9.8 (SD 1.35) [table not provided].

Table 1 shows that after the intervention there was statistically significant reduction in the mean value of HbA1c from 9.8 to 7.7 (*P* < 0.001). Additionally, there was a significant increase in the mean scores of the following HRQoL scales: role limitations due to emotional problems (*P* < 0.05), energy/fatigue (*P* < 0.05), emotional well-being (*P* < 0.001), and general health (*P* < 0.001). Although the mean scores of physical functioning, role limitations due to physical health, social functioning, and pain showed postinterventional improvement, it was not significant (*P* > 0.05).

Table 2 shows that before the intervention the mean ± SD values of HbA1c among the participants of ≤40 and >40 age groups were 9.44 ± 1.38 and 10.03 ± 1.28, respectively, and the difference between them was statistically significant (*P* < 0.05). Additionally, the scores of HRQoL scales were better among participants ≤40 years and the mean scores of five scales (physical functioning, energy/fatigue, social functioning, pain, and general health) were significantly higher (*P* < 0.05) among them than those among patients who were >40 years. After the intervention, the mean values of HbA1c in ≤40 and >40 age groups reduced and became 7.59 ± 0.95 and 7.79 ± 1.09, respectively, with insignificant statistical difference (*P* > 0.05). However, six HRQoL scales showed significant difference between their mean scores in both age groups (*P* < 0.05). Within each group before and after the intervention, it is shown that, among patients who were older than forty years, there is statistically significant increase in the mean scores of the following HRQoL scales: (a) role limitations due to emotional problems (*P* < 0.05), (b) energy/fatigue (*P* < 0.01), (c) emotional well-being (*P* < 0.01), and (d) general health (*P* < 0.05). However, among those who were ≤40 years, there is a significant increase in the mean scores of the following HRQoL scales: (a) energy/fatigue (*P* < 0.05), (b) emotional well-being (*P* < 0.01), and (c) general health (*P* < 0.01).

Table 3 shows that before the intervention the mean ± SD values of HbA1c among males and females were 9.74 ± 1.36 and 9.86 ± 1.35, respectively, with insignificant statistical difference between them (*P* > 0.05). Furthermore, the mean scores of HRQoL scales were better among males than females and the difference between them was statistically significant only in three scales (energy/fatigue, emotional well-being, and general health). After the intervention, despite the reduction in HbA1c and the improvement in the mean scores of HRQoL scales among males and females, the difference between them was statistically insignificant except in the three scales (energy/fatigue, emotional well-being, and general health). Within each group before and after the intervention, it is shown that, among males, there is statistically significant increase in the mean scores of four HRQoL scales (role limitations due to physical health (*P* < 0.05), energy/fatigue (*P* < 0.01), emotional well-being (*P* < 0.001), and general health (*P* < 0.01)). Meanwhile, among females, there is a significant increase in the mean scores of three HRQoL scales (role limitations due to emotional problems (*P* < 0.05), emotional well-being (*P* < 0.05), and general health (*P* < 0.05)).

Table 4 shows that before the intervention the mean scores of HRQoL scales were better among educated than uneducated participants and the difference between them was statistically significant only in two scales (physical functioning, role limitations due to emotional problems). After the intervention, despite the reduction in HbA1c and the improvement in the mean scores of HRQoL scales among educated and uneducated participants, the difference between them was statistically insignificant except in physical functioning scale (*P* < 0.05). Within each group before and after the intervention, there is statistically significant increase in the mean scores in four scales of HRQoL (role limitations due to emotional problems (*P* < 0.05), energy/fatigue (*P* < 0.05), emotional well-being (*P* < 0.01), and general health (*P* < 0.01)).

## 4. Discussion

To the best of our knowledge, this is the first study addressing the effects of a psychoeducational program on HRQoL and HbA1c in patients with type 2 DM in Jazan city. The results revealed that the psychoeducational intervention based on interactive educational approaches such as counseling, demonstration, group discussion, and vignettes improved HbA1c and HRQoL in patients with type 2 diabetes. Improvements in HbA1c and HRQoL might be related to behavior change, and the behavior change could be related to the upgrading of patients' knowledge. It was reported that counseling serves to augment the coping capacity of the patient to address the impact of his illness [17, 26], and the group-based interactive approaches increase the level of knowledge among diabetics [9, 27].

The results revealed that a significant difference existed between HbA1c levels before and after the psychoeducational intervention. These findings of the study are compatible with those of other studies [9, 28, 29]. Reduction of HbA1c is mainly an outcome of the behavior modification of participants. After 12 weeks of educational period, the average of blood glucose levels was closer to normal [30]. Maintaining low levels of HbA1c is essential for preventing DM complications [31].

The QoL improvement for diabetics in a way that they can live a normal life is an aim of diabetes management plans. It is an important outcome measuring method that should be assessed on a routine basis in clinical studies, which are interested in evaluating patients' education [28]. The results indicated that before the intervention patients had lower HRQoL. After the intervention, HRQoL was significantly improved except in physical functioning, physical role, and social and bodily pain scales. Increasing evidence from different studies has also suggested the positive impact of such educational programs on various QoL dimensions [32–34].

The results showed that before the intervention patients who were older than 40 years had lower scores in all HRQoL scales, compared with those who were forty years old or less. These findings are consistent with other studies which found that age is negatively correlated with HRQoL [33–35]. The findings could be interpreted accordingly, such that diabetes complications are prevalent among older diabetics, and they are determinants of poor HRQoL [34].

After the intervention, comparing the means of the outcome variables in both age groups with each other (between groups) revealed that the means of HbA1c and HRQoL scales had been improved among the two groups, but they were still better among patients who were forty years old or less, and the improvement was significant in six HRQoL scales (physical functioning, energy/fatigue, emotional well-being, social functioning, pain, and general health). These findings are consistent with those of other study which found that after educational intervention the HRQoL was better among those who were younger than forty years [36].

Within each group before and after the intervention, the results found that more HRQoL scales showed significant improvement among participants who were older than forty years of age compared with the others. There was a shortage of supporting or contrasting evidences, and the findings could be interpreted with the reasoning that older diabetics usually follow the recommendations of such programs because (a) the majority of them are cautious [37], (b) older people are adaptable [37], and (c) they have a “preference for routine” [37].

Regarding the gender, the results showed that before intervention females had lower scores in most HRQoL scales than males. The difference between males and females was significant in three scales (energy/fatigue, emotional well-being, and general health). This finding is consistent with those of other studies that found that diabetic females have worse HRQoL than males [38, 39]. Another study indicated female gender as an independent risk factor for low HRQoL [35]. After the intervention, the mean values of HbA1c and HRQoL scales improved among males and females. This finding is in agreement with those of other studies which found that after intervention the mean scores of HRQoL scales were higher among males than females [32].

Within each gender group before and after the intervention, the results found that more HRQoL scales showed significant improvement among males after the intervention compared with the case among female participants. These findings reflected a better response of males towards the interventional program and this is consistent with findings of other studies [32, 36]. The variation between males and women in the Saudi community [40] could be explained by the following: (a) most of the women usually spend most of their time in their houses; (b) there is a lack of physical activity among women; (c) contraindicated eating habits are prevalent among Saudi women; and (d) women are more emotional, while males are able to control their diabetes more and are less likely to have psychological problems than women [41]. Generally, each gender has its specific characteristics (physical, mental, and social) that vary between each other [40].

Regarding the education of the participants, the results revealed that the mean value of HbA1c HRQoL scales was better among the educated participants compared with uneducated ones, before and after the intervention program. It may be interpreted that with the education the patients' knowledge and awareness increased in terms of self-care, good compliance with medication, and prevention from DM complications and this could improve QoL of patients. The findings are consistent with the results of studies by Borhani et al. [42] and Glasgow et al. [43].

Within each group before and after the intervention, the results found that both groups (educated and uneducated) had good response to the psychoeducational program. The findings are in agreement with Hossien and Mohammad [32] and Shabibi et al. [36] who reported that, within educated and uneducated groups, HRQoL was significantly improved after the intervention.

The main strengths of this study were as follows: (a) the psychoeducational intervention was practically designed for the conduction and implementation in the primary health care centers; and (b) essential and basic principles related to interactive educational techniques were fully considered in the educational sessions. Our limitations include the following. (a) The sample size is small and follow-up period is short. Therefore, we recommend the use of a large sample size and increased periods of educational follow-up for researchers who are interested in conducting similar psychoeducational interventional studies in the future. (b) As many quasi-experimental studies, the lack of random assignment into test groups leads to nonequivalent test groups which in turn affect the generalizability of the results to a larger population. In addition to that, conclusions on causality are less definitive in quasi-experimental designs. (c) Another potential limitation is uncontrolled confounding which may occur when hidden variables other than the intervention may change over time or differ between preintervention and postintervention period. To overcome this situation the study team identified all known confounders, such as the type of DM treatment, where it was ensured that it was not changed during the course of the study program for all the included patients.

## 5. Conclusion

The results highlighted that the application of a psychoeducational program in educating patients with type 2 diabetes was helpful, valuable, and important. After the intervention, considerable changes have been seen in glycemic control indicators and HRQoL. The study recommends that health care providers use the psychoeducation program as a core element in health care of diabetic patients. Moreover, patients with type 2 diabetes should receive ongoing psychoeducation to maintain the beneficial effects of the intervention.

## Figures and Tables

**Table 1:** Comparison of hemoglobin A1c level and quality of life scales before and after the intervention.

Variable	Before the intervention	5 months after the intervention	*P value*
*HbA1c (mean ± SD)*	9.8 ± 1.35	7.7 ± 1.03	***<0.001*** ^*∗*^
*HRQoL scales (mean ± SD)*			
Physical functioning	46.62 ± 19.68	47.22 ± 20.29	*0.434*
Role limitations due to physical health	47.42 ± 22.96	48.54 ± 23.13	*0.103*
Role limitations due to emotional problems	59.21 ± 24.62	62.77 ± 23.69	***0.003*** ^*∗*^
Energy/fatigue	44.14 ± 14. 85	46.97 ± 13.81	***0.002*** ^*∗*^
Emotional well-being	44.65 ± 13.31	49.05 ± 15.39	***<0.001*** ^*∗*^
Social functioning	57.58 ± 18.02	58.07 ± 17.90	*0.414*
Pain	49.47 ± 24.26	49.91 ± 24.85	*0.500*
General health	50.23 ± 24.14	55.51 ± 24.46	***<0.001*** ^*∗*^

Paired *t*-tests were used in comparing means; ^*∗*^*P* < 0.05 is significant (two-tailed).

**Table 2:** Comparison of the mean ± standard deviation scores of hemoglobin A1c level and quality of life scales regarding the age groups before and after the intervention.

Variables	Before the intervention			5 months after the intervention			*P*^2^	*P* ^3^
	≤40 years	>40 years	*P* ^1^	≤40 years	>40 years	*P* ^1^		
*HbA1c (mean ± SD)*	9.44 ± 1.38	10.03 ± 1.28	***0.030*** ^**∗**^	7.59 ± 0.95	7.79 ± 1.09	*0.327*	**<*0.001*** ^**∗**^	**<*0.001*** ^**∗**^
*HRQoL scales (mean ± SD)*								
Physical functioning	58.17 ± 19.36	38.45 ± 15.45	**<*0.001*** ^**∗**^	59.27 ± 19.70	38.71 ± 16.05	**<*0.001*** ^**∗**^	*0.118*	*0.626*
Role limitations due to physical health	51.10 ± 24.48	44.83 ± 21.66	*0.182*	52.56 ± 24.42	45.69 ± 21.93	*0.146*	*0.103*	*0.105*
Role limitations due to emotional problems	62.58 ± 24.99	56.82 ± 24.29	*0.254*	64.75 ± 21.39	61.36 ± 23.29	*0.485*	*0.087*	***0.010*** ^**∗**^
Energy/fatigue	49.76 ± 13.32	40.17 ± 14.69	***0.001*** ^**∗**^	51.46 ± 13.52	43.79 ± 13.22	***0.006*** ^**∗**^	***0.033*** ^**∗**^	***0.004*** ^**∗**^
Emotional well-being	47.71 ± 13.28	42.48 ± 13.02	*0.054*	52.98 ± 15.30	46.28 ± 14.97	***0.032*** ^**∗**^	***0.002*** ^**∗**^	***0.004*** ^**∗**^
Social functioning	63.72 ± 16.49	53.23 ± 17.92	***0.004*** ^**∗**^	63.71 ± 15.00	54.08 ± 18.80	***0.008*** ^**∗**^	*0.989*	*0.302*
Pain	61.56 ± 22.77	40.93 ± 21.65	**<*0.001*** ^**∗**^	62.33 ± 23.44	41.14 ± 22.06	**<*0.001*** ^**∗**^	*0.569*	*0.726*
General health	57.17 ± 20.05	45.33 ± 25.70	***0.015*** ^**∗**^	63.51 ± 19.14	49.84 ± 26.33	***0.006*** ^**∗**^	***0.003*** ^**∗**^	***0.010*** ^**∗**^

Independent *t*- and paired *t*-tests were used for comparing means, *P*^1^ value between groups, *P*^2^ value within the group (≤40 years) before and after intervention, and *P*^3^ value within the group (>40 years) before and after intervention. ^*∗*^*P* < 0.05 is significant (two-tailed).

**Table 3:** Comparison of the mean ± standard deviation scores of hemoglobin A1c level and quality of life scales regarding the gender before and after the intervention.

Variables	Before the intervention			5 months after the intervention			*P* ^2^	*P* ^3^
	Males	Females	*P* ^1^	Males	Females	*P* ^1^		
*HbA1c (mean ± SD)*	9.74 ± 1.36	9.86 ± 1.35	*0.663*	7.72 ± 1.03	7.69 ± 1.05	*0.892*	**<*0.001*** ^**∗**^	**<*0.001*** ^**∗**^
*HRQoL scales (mean ± SD)*								
Physical functioning	46.05 ± 20.74	47.38 ± 18.35	*0.742*	46.67 ± 21.28	47.98 ± 19.10	*0.753*	*0.253*	*0.391*
Role limitations due to physical health	48.95 ± 22.65	45.36 ± 23.49	*0.445*	50.70 ± 22.75	45.60 ± 23.59	*0.280*	***0.032*** ^**∗**^	*0.323*
Role limitations due to emotional problems	62.49 ± 26.89	54.74 ± 20.64	*0.122*	65.22 ± 26.61	59.43± 18.82	*0.231*	*0.065*	***0.012*** ^**∗**^
Energy/fatigue	47.54 ± 14.55	39.52 ± 14.13	***0.007*** ^**∗**^	50.53 ± 13.29	42.14 ± 13.17	***0.002*** ^**∗**^	***0.004*** ^**∗**^	*0.047*
Emotional well-being	47.02 ± 12.92	41.43 ± 13.31	***0.038*** ^**∗**^	52.70 ± 13.84	44.10 ± 16.16	***0.005*** ^**∗**^	**<*0.001*** ^**∗**^	***0.033*** ^**∗**^
Social functioning	60.09 ± 16.61	54.17 ± 19.46	*0.106*	60.73 ± 16.62	54.45 ± 19.10	*0.085*	*0.487*	*0.675*
Pain	51.89 ± 24.40	46.19 ± 23.96	*0.250*	51.68 ± 25.21	47.52 ± 24.44	*0.414*	*0.822*	*0.093*
General health	54.91 ± 23.53	43.88 ± 23.77	***0.024*** ^**∗**^	61.44 ± 22.38	47.45 ± 25.12	***0.004*** ^**∗**^	***0.001*** ^**∗**^	***0.033*** ^**∗**^

Independent *t*- and paired *t*-tests were used for comparing means, *P*^1^ value between groups, *P*^2^ value within the group (males) before and after intervention, and *P*^3^ value within the group (females) before and after intervention. ^*∗*^*P* < 0.05 is significant (two-tailed).

**Table 4:** Comparison of the mean ± standard deviation scores of hemoglobin A1c level and quality of life scales regarding the education before and after the intervention.

Variables	Before the intervention			5 months after the intervention			*P* ^2^	*P* ^3^
	Educated number = 57	Noneducated number = 42	*P* ^1^	Educated number = 57	Noneducated number = 42	*P* ^1^		
*HbA1c (mean ± SD)*	9.68 ± 1.40	9.93 ± 1.28	*0.376*	7.54 ± 0.85	7.93 ± 1.22	*0.067*	**<*0.001*** ^**∗**^	**<*0.001*** ^**∗**^
*HRQoL scales (mean ± SD)*								
Physical functioning	51.23 ± 19.87	40.36 ± 17.79	***0.006*** ^**∗**^	52.11 ± 20.44	40.60 ± 18.32	***0.005*** ^**∗**^	*0.192*	*0.570*
Role limitations due to physical health	49.30 ± 23.46	44.88 ± 22.29	*0.347*	50.79 ± 23.73	45.48 ± 22.19	*0.261*	*0.055*	*0.168*
Role limitations due to emotional problems	63.75 ± 22.97	53.04 ± 25.69	***0.032*** ^**∗**^	65.88 ± 22.04	58.54 ± 25.41	*0.128*	***0.046*** ^**∗**^	***0.018*** ^**∗**^
Energy/fatigue	45.26 ± 15.37	42.62 ± 14.15	*0.384*	47.89 ± 14.11	45.71 ± 13.46	*0.440*	***0.010*** ^**∗**^	***0.022*** ^**∗**^
Emotional well-being	46.67 ± 14.11	41.90 ± 11.76	*0.078*	50.18 ± 15.68	47.52 ± 15.05	*0.400*	***0.009*** ^**∗**^	***0.001*** ^**∗**^
Social functioning	59.87 ± 17.32	54.46 ± 18.69	*0.141*	60.51 ± 16.68	54.75 ± 19.13	*0.114*	*0.487*	*0.675*
Pain	50.55 ± 25.68	48.01 ± 22.41	*0.609*	51.56 ± 26.51	47.68 ± 22.51	*0.445*	*0.196*	*0.767*
General health	53.04 ± 24.67	46.43 ± 23.15	*0.180*	57.72 ± 24.97	52.50 ± 23.72	*0.296*	***0.005*** ^**∗**^	***0.007*** ^**∗**^

Independent *t*- and paired *t*-tests were used for comparing means, *P*^1^ value between groups, *P*^2^ value within the group (educated) before and after intervention, and *P*^3^ value within the group (noneducated) before and after intervention. ^*∗*^*P* < 0.05 is significant (two-tailed).

## Data Availability

The datasets used and/or analyzed during the present study are available from the corresponding author on reasonable request.
